# P02.21. Short- and long-term effects of expressive writing in patients with renal cell carcinoma

**DOI:** 10.1186/1472-6882-12-S1-P77

**Published:** 2012-06-12

**Authors:** L Cohen, N Tannir, E Jonasch, L Pisters, S Matin, A Spelman, Q Wei, C Wood

**Affiliations:** 1 The University of Texas MD Anderson Cancer Center, Houston, USA

## Purpose

Most previous research examining the efficacy of brief expressive writing interventions have used small sample sizes and followed people for no more than 3 months. We conducted a large randomized trial to examine an emotion-based writing intervention for patients with renal cell carcinoma and followed them for 10 months after the end of the writing sessions.

## Methods

Two hundred patients with renal cell carcinoma were randomly assigned to either an expressive writing group (EW) or a neutral writing group (NW) and asked to write on four separate occasions over 10 days for a maximum of 20 minutes at each writing session. Patients completed the MD Anderson Symptom Inventory (MDASI), Brief Fatigue Inventory, SF-36, IES, CES-D, and PSQI at baseline and then again 1 and 10 months after the writing sessions.

## Results

The mean age of the participants was 58 (range 34-82 years), 41% were women, and 46% had advanced disease. Examination of group differences 1 month after the writing sessions, controlling for the respective baseline measure, revealed lower IES scores for the EW group (intrusive thoughts: EW, 5.0 v NW, 7.2; p<.02; avoidance behaviors: EW, 6.3 v NW, 8.7; p<.07). At the 10 month time point, the EW group reported lower MDASI interference scores (symptoms interfering with QOL) (EW: 6.4 v NW: 9.9; p<.04), higher levels of SF-36 Role Physical scores (EW: 69.6 v NW: 54.0; p<.02), and fewer sleep disturbances (subscale of the PSQI; EW: 1.4 v NW: 1.6; p<.05). Means for the other SF-36 subscales at 10 months were in the expected direction, but did not reach statistical significance. There were no group differences for CES-D or fatigue scores at any time point.

## Conclusion

These findings indicate expressive writing leads to short-term reduction in intrusive thoughts about the cancer experience and results in long-term improvement in aspects of quality of life.

